# Supplementary material for: Association between in situ ventilation and human-generated aerosol exposure in meatpacking plants during the COVID-19 pandemic
Source: PLoS One. 2024 Dec 17;19(12):e0314856. doi: 10.1371/journal.pone.0314856 (PMC11651551; doi:10.1371/journal.pone.0314856)
Supplement: S1 Table — No SARS-CoV-2 was detected in any of the locations sampled. SFTPC was detected in 14 samples distributed across most of the areas. ML = men’s locker room, WL = women’s locker room and C = cafeteria. (PDF) [file pone.0314856.s001.pdf]

Site A	Number of Samples	SARS-CoV-2 Detected	SARS-CoV-2 Concentration	Human Surfactant Detected	Human Surfactant Concentration						CO2 ppm
Processing and Packing Areas (copies/L air)	38	0 samples	NA	5 samples	**mean**	2.4E+01	7.6E+00	6.5E+00	7.6E+03	6.5E+00	2846
		0%	NA	13%	**std. dev.**	4.1E+01	1.3E+01	1.1E+01	7.1E+02	8.9E+00	1118
Harvest Areas (copies/L air)	12	0 samples	NA	2 samples	**mean**	1.9E+01	1.3E+01				2138
		0%	NA	17%	**std. dev.**	2.0E+01	2.3E+01				1519
Cafeterias (copies/L air)	14	0 samples	NA	3 samples	**mean**	1.3E+01	1.8E+01	1.6E+01			1565
		0%	NA	21%	**std. dev.**	2.3E+01	3.1E+01	2.8E+01			323
Common Areas (copies/L air)	15	0 samples	NA	2 samples	**mean**	1.5E+01	3.0E+01				1426
		0%	NA	13%	**std. dev.**	1.3E+01	3.5E+01				
					**Location**	ML	WL				847
Long Term Samples (total copies)	6	0 samples	NA	2 samples	**mean**	3.0E+04	1.2E+04				
		0%	NA	33%	**std. dev.**	3.0E+04	1.1E+04				
					**Location**	ML	C				
