# Supplementary material for: Association between in situ ventilation and human-generated aerosol exposure in meatpacking plants during the COVID-19 pandemic
Source: PLoS One. 2024 Dec 17;19(12):e0314856. doi: 10.1371/journal.pone.0314856 (PMC11651551; doi:10.1371/journal.pone.0314856)
Supplement: S2 Table — No SARS-CoV-2 was detected in any of the locations sampled. SFTPC was detected in 6 samples most of which were in the common areas and cafeterias. ML = men’s locker room, WL = women’s locker room and C = cafeteria. (PDF) [file pone.0314856.s002.pdf]

Site B	Number of Samples	SARS-CoV-2 Detected	SARS-CoV-2 Concentration	Human Surfactant Detected	Human Surfactant Concentration					
Processing and Packing Areas (copies/L air)	36	0 samples	NA	0 samples	**mean**					1160.924
		0%	NA	0%	**std. dev.**					148.5414
Harvest Areas (copies/L air)	12	0 samples	NA	1 sample	**mean**	9.4E+00				530.0417
		0%	NA	8%	**std. dev.**	1.6E+01				52.00211
Cafeterias (copies/L air)	12	0 samples	NA	1 sample	**mean**	5.8E+00				689.25
		0%	NA	8%	**std. dev.**	1.0E+01				104.6832
Common Areas (copies/L air)	12	0 samples	NA	4 samples	**mean**	5.2E+00	3.5E+00	4.2E+00	3.8E+00	800.25
		0%	NA	33%	**std. dev.**	8.9E+00	6.0E+00	7.3E+00	6.6E+00	
					**Location**	MH	MH	ML	WL	162.845
Long Term Samples (total copies)	6	0 samples	NA	0 samples	**mean**					
		0%	NA	0%	**std. dev.**					
