# Supplementary material for: Association between in situ ventilation and human-generated aerosol exposure in meatpacking plants during the COVID-19 pandemic
Source: PLoS One. 2024 Dec 17;19(12):e0314856. doi: 10.1371/journal.pone.0314856 (PMC11651551; doi:10.1371/journal.pone.0314856)
Supplement: S3 Table — SARS-CoV-2 was detected in both long-term (1) and discrete term air samples (1). SFTPC was detected in 18 samples distributed across most of the areas. ME = Main Entry, MH = Main Hallway, ML = men’s locker room, WL = women’s locker room and C = cafeteria. (PDF) [file pone.0314856.s003.pdf]

Site C	Number of Samples	SARS-CoV-2 Detected	SARS-CoV-2 Concentration	Human Surfactant Detected	Human Surfactant Concentration							CO2 ppm
Processing and Packing Areas (copies/L air)	30	0 samples	NA	5 samples	**mean**	5.3E+00	7.0E+00	2.3E+01	9.1E-01	5.2E+00		5257.146
		NA	NA	17%	**std. dev.**	9.1E+00	1.2E+01	3.9E+01	1.6E+00	9.0E+00		1498.728
Harvest Areas (copies/L air)	30	0 samples	NA	4 samples	**mean**	3.8E+00	8.2E+00	3.0E+00	2.8E+00			562.661
		0%	NA	13%	**std. dev.**	6.5E+00	1.4E+01	5.1E+00	4.9E+00			94.60747
Cafeterias (copies/L air)	15	0 samples	NA	3 samples	**mean**	3.1E+00	4.1E+00	7.8E-01				1028.769
		NA	NA	20%	**std. dev.**	5.5E+00	7.2E+00	1.4E+00				852.847
Common Areas (copies/L air)	15	0 samples	NA	6 samples	**mean**	7.9E+00	2.1E+00	3.5E+00	1.6E+01	1.2E+00	7.2E-01	1504.125
		NA	NA	40%	**std. dev.**	7.0E+00	3.6E+00	6.1E+00	1.5E+01	2.0E+00	1.3E+00	
					**Location**	ME	MH	ML	ME	MH	WL	2351.405
Long Term Samples (total copies)	7	1 samples	9.7E+05	5 samples	**mean**	2.9E+03	1.0E+05	6.8E+03	7.2E+03	1.7E+04		
		14%	4.37E+05	71%	**std. dev.**	2.5E+03	1.0E+05	1.2E+04	6.5E+03	1.9E+04		
		**Location**	ML		**Location**	WL	ML	MH	ME	C		
