# Supplementary material for: Association between in situ ventilation and human-generated aerosol exposure in meatpacking plants during the COVID-19 pandemic
Source: PLoS One. 2024 Dec 17;19(12):e0314856. doi: 10.1371/journal.pone.0314856 (PMC11651551; doi:10.1371/journal.pone.0314856)
Supplement: Attachment — Submitted filename: Response to Reviewers_final.docx [file pone.0314856.s004.docx]

Response to Reviewers

We appreciate the time and effort by the reviewer to provide comments on our manuscript. We have carefully considered each comment and have provided a point-by-point response (in red) to each comment. We have also included a track changes version of manuscript, along with the clean version.

Reviewer #1: Thank you for this overall nicely written and relevant manuscript. I think it is an approach to discuss which working areas are most exposed to SARS-CoV-2. Find attached some general and specific comments:

1) As someone who has closely followed the outbreak events in meat plants, I realized that in my country meat factories are very heterogeneous depending on the focus. Your sample consists of three facilities from America and I also had the feeling that the plants there are very heterogeneous. Is it possible to generalize the results in this way?

The reviewer raises an important point regarding the heterogeneity of the meatpacking facilities worldwide. As described in the manuscript, Sites A and C were similar in size, scale and ventilation, while Site B was significantly newer, processed a different protein (poultry, rather than beef) and had much different work practices. The conclusions regarding increased airflow minimizing exposure to human-generated aerosol were generated by synthesizing the data from all 3 facilities. The conclusion that under ventilated spaces represent a greater risk of exposure is not unique to this study, but the data gathered here strongly support that increasing ventilation reduces the exposure to human-generated aerosols that may transmit disease.

2) Data availability & Methods & Statistical part - you are reporting a lot of parameter like mean, standard deviation, correlation (,etc.?) and for me it was unclear how you calculated these estimates. Could you explain this in more detail in the methods section? Otherwise I can't judge whether you have really disclosed all the data, especially for the mean values.

We originally assumed that the calculated means and standard deviations were sufficient in our data disclosure. Based on the reviewers comments, we are now posting all raw data to the following data repository: Santarpia, Joshua, 2024, "Replication Data for: Association between in situ ventilation and human-generated aerosol exposure in meatpacking plants during the COVID-19 pandemic", <https://doi.org/10.7910/DVN/J6BQES>, Harvard Dataverse

3) Could you provide more information about the measurements of each of the tests in the methods section? How and when were the measurements carried out? Were all measurements long-term measurements or were individual measurements only carried out once. This would have to be specified here. Were all measurements in real life situations or were some measurements in empty rooms?

Due to the release of higher concentrations of CO2 in Test 1, the test was carried out during no occupancy. Tests 2, 3, 4 and 5 were carried out during normal work, when the spaces were occupied. Test 5, in particular, was carried out during periods of maximum occupancy (page 10 in the test 5 description). Test 1 was performed according to a standard method (ASTM D6245), Test 2 was calculated based on 5 successive measurements (as described in the text, Page 8). As described in the text, the ventilation for Test 3 was calculated based on measurements over 5 consecutive days. The number of individual samples for Tests 4 and 5 are described in Table 2, as well as in Table 5 and all supplemental tables. Test 4 was the only multi-day measurement, where single samples were collected over periods of several days (as described in the text). For tests 3 and 5, short duration measurements were made repeatedly over several days. The descriptions of the tests have been updated (where not explicitly stated) to reflect the above description.

4) Something went wrong with the tables and there are two different tables 1. Formatting is also different here. This made it really difficult to follow the text. Table numbering (from Table 4 onwards) is therefore incorrect.

We appreciate the reviewer identifying this issue. The original manuscript version had many tables in the text that were moved to the supplement during editing. There were some residual incorrect figure references, as well as misnumbered tables that we did not catch prior to submission. The tables have now been correctly numbered and referenced in the text.

5) In the part "Assay of Samples" I have the impression that methods are here in the result section. Unfortunately, I cannot really assess the results here and if results are presented in this way, the comment can be ignored.

The “Assay of Samples” is a subsection of the Methods. It describes, in detail, how each of the samples collected in the preliminary study of Site A and Tests 4 and 5 were assayed for the presence of SARS-CoV-2 and SFTPC, how the copy number is calculated from the raw Ct value, and how the copy number is converted to relevant environmental concentration units, based on how each sample was collected. The resulting calculated data is presented in the Tables and Figures in the text and supplement.

6) I would structure the discussion differently. First summarize the most important results and then in the strengths section you can go into why the study is special. You can also take a look at the recommendations in the strobe statement (<https://www.strobe-statement.org/>).

We have worked to restructure the discussion section according to the reviewer’s recommendations.

7) Source 19 looks a bit strange (Herstein J, SM, LA, DE, RA, GB, B-MD, KC, LJ, & LJ.). Overall, the bibliography should be standardized again.

We thank the reviewer for catching this error. There were some import errors in the reference manager software, which we have now corrected.
