# Supplementary material for: Association between in situ ventilation and human-generated aerosol exposure in meatpacking plants during the COVID-19 pandemic
Source: PLoS One. 2024 Dec 17;19(12):e0314856. doi: 10.1371/journal.pone.0314856 (PMC11651551; doi:10.1371/journal.pone.0314856)
Supplement: Attachment — Submitted filename: Response to Reviewersv2_final.docx [file pone.0314856.s005.docx]

Response to Reviewers

We appreciate the time and effort by the reviewer to provide comments on our manuscript. We have carefully considered each comment and have provided a point-by-point response (in red) to each comment. We have also included a track changes version of manuscript, along with the clean version.

Reviewer #1: Thank you for incorporating my comments. I have to ask again about point two. Could you include which software was used to perform the calculations? If all the calculations have already been done by the measuring devices, the description of how the tables and figures were created is also sufficient. Finally, I can only find one error in Table 6, where the table labeling is strange.

We appreciate the reviewers clarification of their previous comment. In response, we have added a short section at the end of the methods section entitled “Data tabulation and Analysis”, which addresses which software packages were utilized, and in what way for the analysis of the data.

We have attempted to clarify the titles in Table 6 to ensure that they are clear.
